# The role of *Moringa oleifera* in enhancing fish performance and health: a comprehensive review of sustainable aquaculture applications

**DOI:** 10.1007/s11259-025-10889-4

**Published:** 2025-09-10

**Authors:** Sohair Y. Saleh, Nehal A. Younis, Ahmed H. Sherif, Howyda G. Gaber

**Affiliations:** 1 Department of Physiology, Faculty of Veterinary Medicine, Cairo University, PO 11221, Giza, Egypt; 2 Department of Aquatic Animal Medicine and Management, Faculty of Veterinary Medicine, Cairo University, Giza, Egypt PO 11221,; 3 Fish Disease Department, Animal Health Research Institute AHRI, Agriculture Research Center ARC, PO 12619, Kafrelsheikh, Egypt

**Keywords:** *Moringa Oleifera*, Aquaculture, Feed additive, Growth performance, Immunostimulant, Disease resistance, Water clarification, Sustainable aquaculture, Economic analysis

## Abstract

This comprehensive review examines the versatile applications and effects of *Moringa oleifera* across multiple fish species in aquaculture systems amid growing challenges of rising feed costs and antimicrobial resistance. *M. oleifera*, commonly called the Miracle tree, contains an exceptional nutritional profile with high protein content (22.99–29.36% dry weight), complete profiles of essential amino acids, and remarkably elevated levels of vitamins and minerals. Its bioactive profile includes substantial quantities of polyphenols, flavonoids, alkaloids (niazimicin, niaziminin, β-sitosterol), and glucosinolates that collectively enhance fish health and performance. Recent studies across multiple fish species demonstrate variable optimal inclusion rates (5–30%) with specific growth rates increasing 15–35% and feed conversion ratios improving 10–25% compared to controls. Physiological benefits include enhanced digestive enzyme activity, improved liver and kidney functions, and reduced cortisol levels during environmental stress. Immunologically, *M. oleifera* supplementation significantly increases lysozyme activity, complement C3, total IgM levels, and pro-inflammatory cytokine expression (TNFα, IL1-β, IL-6), strengthening resistance against major aquaculture pathogens including *Aeromonas hydrophil*a, *Pseudomonas aeruginosa*, and *Vibrio* species. Challenge studies demonstrate relative percent survival improvements ranging from 48 to 73% against virulent pathogens. Haematological improvements include increased erythrocyte counts, higher haemoglobin levels at moderate inclusion rates, and beneficial effects on glucose metabolism and lipid profiles. Moringa seeds are highly effective natural water clarifiers, removing 95–99% of turbidity through cationic proteins that act as coagulants. However, their antinutritional compounds (tannins, phytates, saponins, oxalates) can harm fish at elevated levels, requiring careful dosage monitoring in aquaculture applications. This review synthesizes current evidence, identifies optimal application strategies, and provides direction for future research to maximize Moringa’s potential as a sustainable, cost-effective resource for enhancing productivity, fish health, and environmental sustainability in modern aquaculture systems.

## Introduction

Aquaculture has emerged as a vital global industry, providing essential food security to a growing global population and playing a significant role in supplying affordable animal protein. Global finfish aquaculture production reached 223.2 million tonnes in 2022, making it one of the fastest-growing food production systems **(**FAO 2024**).** However, the aquaculture industry faces critical sustainability and economic challenges. Fishmeal costs have surged due to declining wild fish stocks, competition from livestock sectors, and climate impacts on marine ecosystems. Feed expenses now dominate operational costs in intensive systems, creating urgent demand for sustainable alternatives as global aquaculture feed requirements continue expanding rapidly **(**Fantatto et al. 2024). Simultaneously, the emergence of antimicrobial resistance in aquaculture pathogens has created an urgent need for natural alternatives to synthetic antibiotics. Resistance rates among major fish pathogens have increased substantially across major aquaculture regions. These challenges necessitate exploration of sustainable, cost-effective alternatives for both nutrition and health management in aquaculture systems **(**Sherif et al. 2022,2023; Hussein et al. 2023; Abd El-Naby et al. 2024).

Multiple fish species contribute significantly to global aquaculture output, including African sharptooth catfish (*Clarias gariepinus*), Nile tilapia (*Oreochromis niloticus*), common carp (*Cyprinus carpio*), and rainbow trout (*Oncorhynchus mykiss*). These species collectively represent over 60% of global finfish production and serve diverse markets from subsistence farming to commercial operations worldwide **(**Naylor et al. 2021). Aquaculture fish species play crucial roles in global food security, with production systems ranging from extensive pond culture to intensive recirculating aquaculture systems, where bacterial disease outbreaks can result in high mortality rates across all major species **(**Wise et al. 2021; Ouma and Barasa 2022; Okasha et al. 2025a, b; Farag et al. 2024; Tawfeek et al. 2024). Modern aquaculture systems face significant challenges across all major fish species, particularly in developing cost-effective, high-quality feeds while preventing disease outbreaks that threaten production stability. Common pathogens affecting multiple species include *A. hydrophila*, *Vibrio* spp, *Streptococcus* strains, and *Flavobacterium* species, creating substantial treatment expenses across different production systems **(**Sherif and Zommara 2024; Basry et al. 2024).

**Fig. 1:**
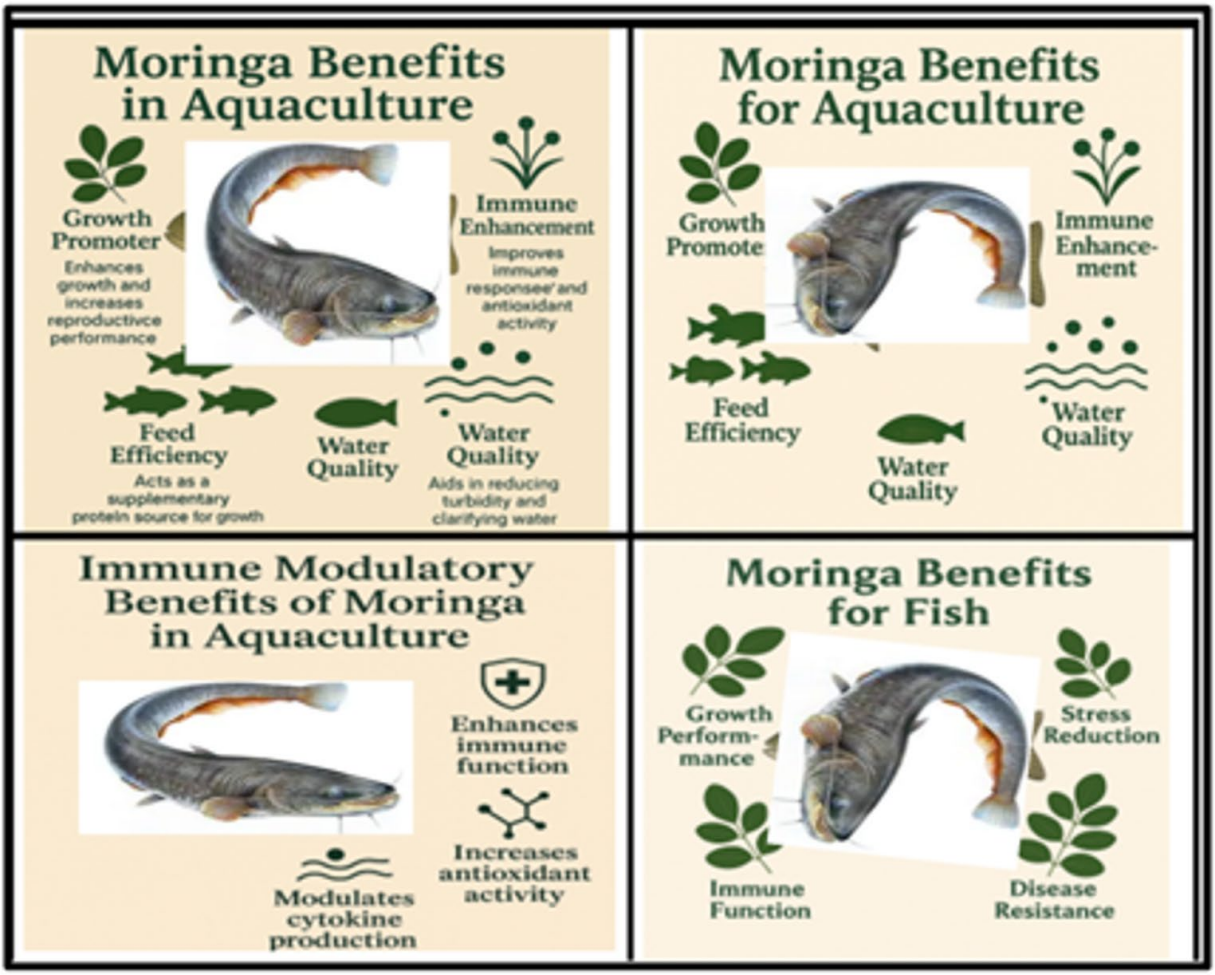
*M. oleifera* Benefits in Aquaculture

**Fig. 2:**
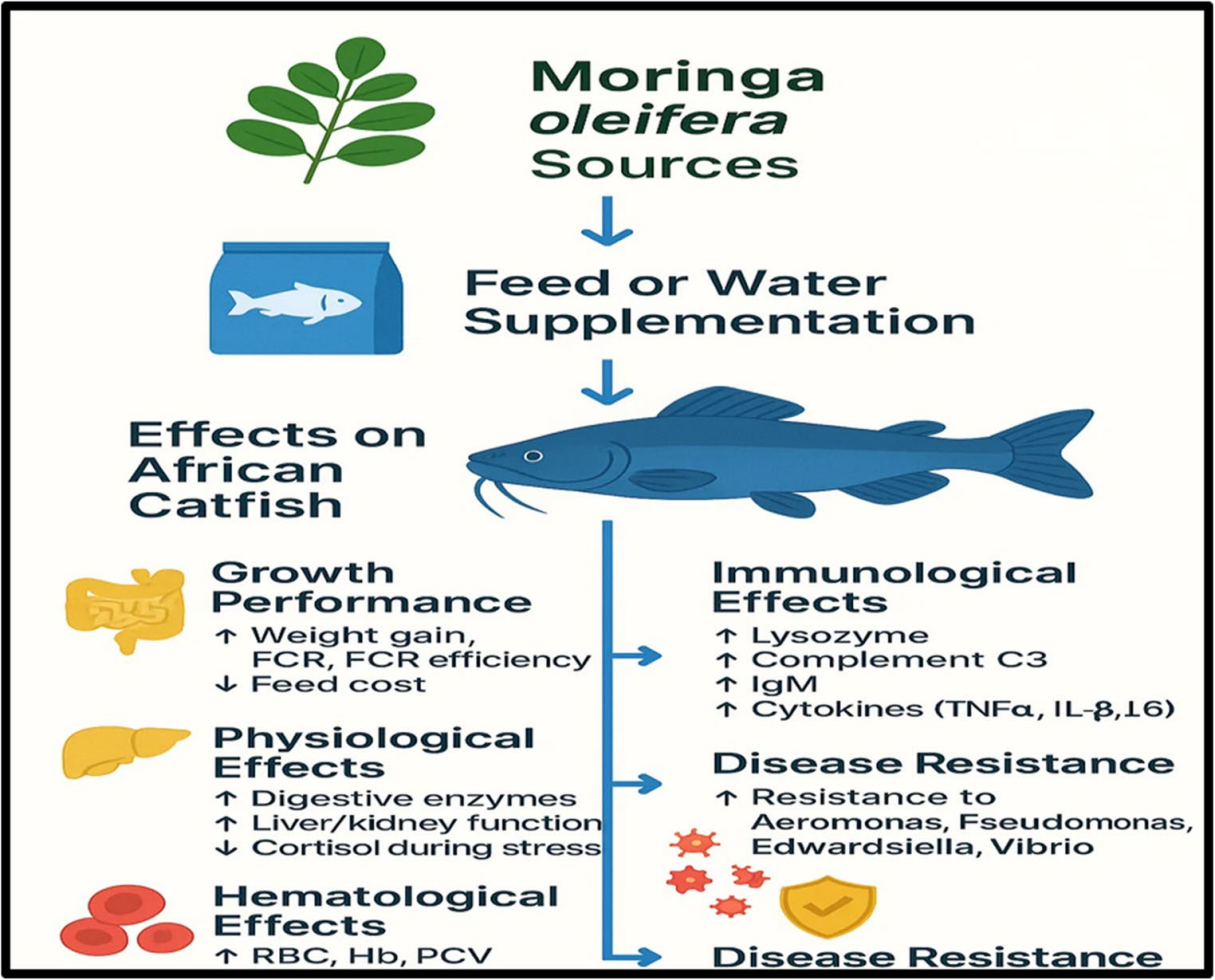
Effects of *M. oleifera* on African Catfish

Among these, *Moringa oleifera* has emerged as a promising plant-based candidate due to its exceptional nutritional profile, and remarkable immunostimulatory and antimicrobial properties **(**Khalil and Korni 2017; Abdelkader et al. 2025). *M. oleifera*, often referred to as the “Miracle tree,” is a fast-growing, drought-resistant tree native to the Indian subcontinent but now cultivated throughout tropical and subtropical regions. The use of *M. oleifera* dates back to ancient civilizations; Egyptians utilized *M. oleifera* oil as a sunscreen **(**Abdelazim et al. 2024), while the ancient Greeks discovered its medicinal properties and introduced it to the Romans, facilitating its spread throughout Europe **(**Abdelazim et al. 2024). This plant has gained significant attention in recent years due to its remarkable nutritional and therapeutic properties, with various parts of the plant—particularly the leaves and seeds—being utilized for different purposes (Figs. 1 and 2). *M. oleifera* leaves are exceptionally rich in essential nutrients, containing elevated levels of proteins, vitamins, minerals, and bioactive compounds (Table 1) **(**Rhyem 2018**).** Recent studies have extensively investigated *M. oleifera* applications across multiple fish species, with systematic analysis revealing species-specific responses and optimal inclusion levels (Table 2). The seeds have garnered interest for their water clarification properties (Alam et al. 2020). However, concerns exist regarding their possible toxic effects on fish health **(**Mahaveerchand and Abdul Salam 2024; Moustafa and Assem 2024**).**

**Table 1:** Nutritional composition of M. oleifera leaves powder (Per100 g)

Compound	Amount	
Calories	205 kcal	
Protein	27.1 g	
Fat	2.3 g	
Carbohydrate	38.2 g	
Fiber	19.2 g	
Calcium (Ca)	2,003 mg	
Magnesium (Mg)	368 mg	
Phosphorous (P)	204 mg	
Potassium (k)	1,324 mg	
Iron (Fe)	28.2 mg	
Vit. A (*β* carotene)	163 mg	
Vit.B_1_ (Thiamine)	2.6 mg	
Vit.B_2_ (Riboflavin)	20.5 mg	
Vit.B_3_ (Niacin)	8.2 mg	
Vit. C	17.3 mg	
Vit. E		113.0 mg

**Table 2:** Comprehensive summary of previous studies on *M. oleifera* in aquaculture

Species	M. oleifera Form	Inclusion Level (%)	Initial Weight (g)	Final Weight (g)	SGR (%/day)	FCR	Key Findings	References
*O. niloticus*	Leaf extract	0, 0.5, 1, 1.5, 2%	32.4 ± 0.1	61.2 ± 1.2	28.8 ± 1.2	1.45 ± 0.03	Enhanced *Streptococcus* resistance	Kamble et al. (2024)
*H. fossilis*	Leaf meal	0, 10, 20, 40%	1.58 ± 0.05	5.62 ± 0.20	1.47 ± 0.04	1.40 ± 0.05	10% inclusion optimal	Sharker et al. (2024)
*C. mrigala*	Leaf meal	0, 10, 15, 20, 25, 30%	15.35 ± 0.04	422.32 ± 0.06	-	-	Enhanced hematological parameters	Faisal et al. (2024)
*O. niloticus*	Aqueous extract	0, 100, 200, 400 mg/kg	7.28 ± 0.31	63.74 ± 1.84	1.76 ± 0.03	1.45 ± 0.09	200 mg/kg optimal, 400 mg/kg toxic	Emam et al. (2024)
*O. niloticus*	Fermented leaves	0, 5, 10%	30.00 ± 0.50	45.00 ± 0.23	1.40 ± 0.03	-	Fermentation improved efficacy	Nassar et al. (2024)
*O. mykiss*	Leaf meal	0, 4, 8, 16%	1805.89 ± 77.79	2392.89 ± 87.76	0.30 ± 0.03	-	8% optimal for reproduction	Momin and Memiş (2023)
*C. auratus gibelio*	Fermented leaves	0, 20, 40, 60%	19.37 ± 0.04	39.51 ± 0.53	1.44 ± 0.03	-	Enhanced *Aeromonas* resistance	Zhang et al. (2020)
*C. gariepinus*	Leaf meal	0, 10, 20, 30	2.20 ± 0.28	6.72 ± 0.15	-	8.19 ± 0.01	30% inclusion optimal for growth	Adekilekun et al. (2022)
*O. niloticus*	Leaf supplement	0, 4, 8, 10%	45.21 ± 30.16	90.42 ± 1.8	1.59 ± 1.30	1.52 ± 0.06	Improved gut microbiota	Parveen et al. (2021)
*Penaeus vannamei*	Leaf extract	0, 1.25, 2.5, 5 g/Kgm	0.54 ± 0.01	6.96 ± 0.47	4.26 ± 0.11	1.29 ± 0.01	Enhanced *Vibrio* resistance	Abidin et al. (2021)
*O. niloticus*	Leaf powder	0, 5, 10%	28.42 ± 0.80	48.67 ± 2.59	2.83 ± 0.09	2.84 ± 0.20	Improved lipid profile and growth	El-Kassas et al. (2020)
*C. idella*	Leaf meal	0, 50, 100, 150 gm/Kgm	21.32 ± 1.440	29.06 ± 1.558	0.3116 ± 0.053	-	Cytokine expression improved	Faheem et al. (2020)
*O. niloticus*	Leaf meal	0, 1.5%	2.0 ± 0.5	20.0 ± 0.7	3.4 ± 0.0	0.04 ± 0.0	Enhanced stress resistance	Elabd et al. (2019)
*O. mossambicus*	Leaf-based diet	0, 3, 6, 9, 12%	33.56 ± 0.94	58.22 ± 4.35	1.71 ± 0.31	2.39 ± 0.28	Enhanced *Aeromonas* resistance	Mbokane and Moyo (2018)

## *M. oleifera*: botanical and nutritional profile

### Botanical classification and geographical distribution

*M. oleifera* Lam., commonly known as the Miracle tree” **(**Biswas et al. 2012), is a fast-growing, drought-resistant species belonging to the Moringaceae family, with a taxonomic classification in the Kingdom Plantae, Order Capparales **(**Pareek et al. 2023). Native to the Himalayan foothills but now cultivated worldwide in tropical and subtropical regions as in many African countries. This versatile tree reaches heights of 10–12 meters with distinctive tripinnate leaves, fragrant white flowers, and elongated triangular seed pods **(**Abdelazim et al. 2024).

#### Standardized processing protocols for aquaculture applications

Standardized processing protocols are essential for consistent *M. oleifera* quality in aquaculture applications (Gopalakrishnan et al. 2016; Abd El-Hack et al. 2022). Raw material preparation involves harvesting fresh *M. oleifera* leaves at 45−60 days maturity **(**Sultana 2020; Pareek et al. 2023), with authentication by certified botanical institutions and quality standards including moisture content < 8% **(**Matthew et al. 2022; Grosshagauer et al. 2021) and heavy metal limits (Pb < 2 mg/kg, Cd < 0.1 mg/kg) according to international feed safety standards **(**Matthew et al. 2022). The standardized cleaning and drying protocol include washing with distilled water (3× rinse cycles) followed by air-drying at 60 ± 2 °C for 24 h **(**Dhakad et al. 2019; Islam et al. 2021) using laboratory oven (Memmert Model UF110, Germany) with continuous temperature monitoring using calibrated data loggers (Onset HOBO MX2301, USA) **(**AOAC 2019**).** Size reduction utilizes hammer mill (Retsch SM 300, Germany) with 0.5 mm mesh, followed by particle size analysis ensuring 90% passing 0.5 mm mesh (Malvern Mastersizer 3000, UK) **(**Oyeyinka and Oyeyinka 2018; Sultana 2020**).** Chemical reagents for analysis include Kjeldahl catalyst tablets (Sigma-Aldrich, Cat. No. 1.09201.0500) for protein analysis **(**AOAC 2019**)**, analytical grade petroleum ether (Fisher Scientific, Cat. No. P/1760/17) for fat extraction **(**AOAC 2019**)**, and Folin-Ciocalteu reagent (Sigma-Aldrich, Cat. No. F9252) for phenolic content determination **(**Vergara-Jimenez et al. 2017; Al-Taweel and Al-Anbari 2019).

### Nutritional composition of leaves

*M. oleifera* leaves represent an exceptional nutritional resource, containing a remarkable profile of essential nutrients and bioactive compounds. The protein content of *M. oleifera* leaves ranges from 22.99−29.36% on a dry weight basis **(**Sultana 2020**)**, with fresh leaves containing 6.7 g/100 g, dry leaves 29.4 g/100 g, and leaf powder 27.1 g/100 g of protein **(**Islam et al. 2021). The leaves incorporate majority of essential amino acids with particularly elevated levels of methionine, cysteine, tryptophan, and lysine, making them a valuable alternative protein source for aquaculture feeds **(**Zaku et al. 2015; Oyeyinka and Oyeyinka 2018**)**. The protein content can vary significantly based on geographical location, soil conditions, and cultivation methods, with studies showing variations even between different districts within the same country **(**Sultana 2020**).** These leaves demonstrate impressive vitamin content, containing approximately 7 times more vitamin C than oranges (fresh leaves: 220 mg/100 g), 10 times more vitamin A than carrots (β-carotene: 16.3 mg/100 g), and substantial amounts of B-complex vitamins including thiamine (B1: 0.06 mg/100 g), riboflavin (B2: 0.05 mg/100 g), niacin (B3: 0.8 mg/100 g), and vitamin E (448 mg/100 g in fresh leaves) **(**Islam et al. 2021; Mushtaq et al. 2021).

The mineral profile of *M. oleifera* leaves further enhances their nutritional value, providing approximately 17 times more calcium than milk (fresh leaves: 440 mg/100 g), 15 times more potassium than bananas (fresh leaves: 259 mg/100 g), 25 times more iron than spinach, and significant concentrations of magnesium (42 mg/100 g), phosphorus (70 mg/100 g), and zinc (3.29 mg/100 g) **(**Islam et al. 2021; Mushtaq et al. 2021). These minerals play critical roles in various physiological processes in fish, including skeletal development, osmoregulation, enzyme activation, and immune function **(**Lall and Kaushik 2021**).** The proximate analysis also revealed that *M. oleifera* leaves contain a relatively low-fat content—fresh (1.7 g/100 g), dry (5.2 g/100 g), and powder (2.3 g/100 g)—and a moderate amount of fiber: fresh (0.9 g/100 g), dry (12.5 g/100 g), and powder (19.2 g/100 g). The carbohydrate content is considerable: fresh (12.5 g/100 g), dry (41.2 g/100 g), and powder (38.2 g/100 g) (Islam et al. 2021).

The bioactive compound profile of *M. oleifera* leaves contributes significantly to their therapeutic properties and potential applications in aquaculture. Polyphenols represent a major group of bioactive compounds in *M. oleifera* leaves, classified as phenolic acids (containing a single phenol ring) and flavonoids (containing multiple phenol rings) **(**Chiș et al. 2023). Flavonoids constitute the primary bioactive compounds in *M. oleifera* leaves, with quercetin, iso-quercitrin, and kaempferol being the most abundant **(**Mali et al. 2022). These compounds exhibit potent antioxidant effects by neutralizing reactive oxygen species (ROS), inhibiting various enzymes involved in oxidative processes, and chelating metals that participate in radical chain reactions, thereby protecting fish against oxidative stress induced by environmental stressors or pathogen exposure **(**Khalil and Korni 2017; Dhakad et al. 2019). The combination of antioxidants in *M. oleifera* leaves is more effective than individual antioxidants, due to synergistic effects and enhanced antioxidant mechanisms **(**Vergara-Jimenez et al. 2017). Phenolic acids identified in *M. oleifera* leaves include gallic acid, salicylic acid, chlorogenic acid, and gentisic acid, which have been extensively studied for their broad-spectrum biological activities, including anti-mutagenic, anti-inflammatory, anti-cancer, and antioxidant properties that can enhance fish health and disease resistance **(**Divya et al. 2024; Gad et al. 2024). Total phenolic content has reported at 785.5 mg/100 g gallic acid equivalent, with total flavonoids at 257 mg/100 g quercetin equivalent, and total antioxidant capacity of 1701.8 mg/100 g ascorbic acid equivalent (Islam et al. 2021; Al-Taweel and Al-Anbari 2019).

Glucosinolates and their hydrolysis products, isothiocyanates, represent another important class of bioactive compounds in *M. oleifera* leaves, with 4-(α-L-rhamnopyranosyloxy)-benzylglucosinolate (glucomoringin) being the predominant glucosinolate **(**Lopez-Rodriguez et al. 2020). Multiple researchers have demonstrated the hypoglycaemic, anti-inflammatory, anti-cancer, and antioxidant effects of these compounds through in vitro and in vivo studies, suggesting potential benefits for fish metabolism and immune function **(**Lopez-Rodriguez et al. 2020). Alkaloids present in *M. oleifera* leaves, including niazimicin, niaziminin, and β-sitosterol, possess notable pharmacological properties such as analgesic, anti-inflammatory, antimicrobial, and antimalarial activities that may contribute to enhanced disease resistance in fish **(**Mali et al. 2022). Additionally, *M. oleifera* leaves contain various terpenes, including all-E lutein, all-E-luteoxanthin, 13-z-lutein, 15-z-β-carotene, and all-E-zeaxanthin, which exhibit diverse biological properties including antifungal, antiviral, antimicrobial, anti-inflammatory, antiparasitic, antihyperglycemic, anti-cancer, and analgesic effects **(**Mali et al. 2022; Lowe et al. 2024). Given their notable medicinal properties, *M. oleifera* leaves can aid in treating various animal diseases. Their potential applications range from serving as antibacterial agents to enhancing immune responses **(**Coppin et al. 2013). Additionally, research has confirmed that crude leaf powder and extracts of *M. oleifera* are effective against *Staphylococcus aureus*,* Bacillus subtilis*, and *Vibrio cholerae* **(**Jayawardana et al. 2015).

Beyond nutritional content, *M. oleifera* leaves also possess valuable functional properties, with water absorption capacity ranging from 158.00 to 415.50%, foaming capacity between 28.30−117.65 mL/L, and foaming stability ranging from 333.33−1000 mL/L after two hours **(**Sultana 2020**).** These functional properties enhance the potential applications of Moringa leaf powder in various food and feed formulations. Due to this exceptional nutritional profile, Moringa has gained recognition as a “Miracle tree” and increasingly incorporated into various food products including soups, paneer, chocolate, biscuits, bread, and muffins **(**Islam et al. 2021). The complex phytochemical profile of Moringa leaves, characterized by synergistic interactions among various bioactive compounds, positions them as promising natural feed additives for enhancing fish growth, health, and disease resistance in sustainable aquaculture systems **(**Hridoy et al. 2025).

### Nutritional composition of seeds

The seeds of *M. oleifera* are especially rich in nutrients, surpassing the nutritional value of its leaves and pods in certain aspects, making them nutritional powerhouse. The seeds consumed either green or dry, adding versatility to their nutritional applications in various food systems Beyond their basic nutritional composition, Moringa seeds contain water-soluble, positively charged proteins that serve as natural coagulants for water purification, making them valuable in areas with limited access to clean water **(**Islam et al. 2021). Ethanol extracts of these seeds have identified seven significant bioactive compounds that contribute to their medicinal properties including 4(α-L-rhamnosyloxy)-benzyl isothiocyanate, niazimicin, 3-O-(6 ^′^-oleoyl-*β*-D-glucopyranoyl)-*β*-sitosterol, *β*-sitosterol-3-O-*β*-D-glucopyranoside, niazirin, *β*-sitosterol, and glycerol-1-(9-octadecnoate), which contribute to their medicinal properties **(**Islam et al. 2021). However, *M. oleifera* seeds also contain anti-nutritional factors such as tannins, phytates, saponins, and cyanogenic glycosides, **(**Matthew et al. 2022). These necessitate careful processing to minimize potential adverse effects. Due to their rich nutritional and bioactive properties, interest in incorporating *M. oleifera* seeds and leaves into aquaculture applications continues to grow.

## Benefits of *M. oleifera* leaves in aquaculture

### Growth performance effects

Leaves of *M. oleifera* are a nutritional powerhouse, abundant in proteins, vitamins, fatty acids, and minerals, often called a “wonder plant,” which has been extensively investigated as a potential substitute for fishmeal in the diets of various fish species, including Nile tilapia, African catfish, and others **(**Mehdi et al. 2016). El-Kassas et al. (2020) reported that 5% *M. oleifera* leaves in the feed significantly lowered the lipid profile and en hanced growth performance.

The remarkable growth-promoting effects of *M. oleifera* have been well-documented across multiple aquaculture species, with particularly promising results in African catfish, where Adekilekun et al. (2022) demonstrated that incorporating up to 30% *M. oleifera* leaf meal in the diet was both feasible and beneficial for maximizing catfish growth, suggesting this level as the most effective for optimal performance while significantly reducing feeding costs and maintaining nutritional health of the fish.

Feed conversion ratio (FCR), a critical parameter in assessing feed efficiency, has shown notable improvements when *M. oleifera* is incorporated into fish diets, as evidenced by Elabd et al. (2019), who reported that including 1.5% *M. oleifera* in the diet of Nile tilapia for three months significantly improved key growth parameters, including body weight gain, length gain, specific growth rate, and feed conversion ratio, while also enhancing several somatic indices that indicate improved overall body condition. This improvement in feed utilization efficiency could be attributed to the bioactive compounds present in *M. oleifera* leaves, which may enhance the activity of digestive enzyme such as amylase, lipase, and proteases like pepsin, trypsin, and chemotrypsin as well as nutrient absorption **(**Guan et al. 2024), while studies have also indicated that *M. oleifera* leaf powder significantly improved the intestinal structure, particularly the length of the intestinal villi, which could be associated with enhanced growth **(**Mahmoud et al. 2022).

Weight gain parameters have consistently shown positive responses to *M. oleifera* supplementation, with Kamble et al. (2024) finding that among various diets tested, the group supplemented with 0.5% *M. oleifera* leaf extract exhibited significantly better growth performance, feed efficiency, blood health indicators, and innate immune responses in Nile tilapia compared to the control group. Specific growth rate (SGR), which measures the percentage of body weight gain per day, has been shown to improve with *M. oleifera* supplementation, with El-Kassas et al. (2020) demonstrating that feeding Nile tilapia fingerlings diets containing *M. oleifera* leaf powder enhanced their growth and improved their blood lipid levels, suggesting that *M. oleifera* leaf powder can be a valuable addition to their feed, potentially replacing other commonly used feed ingredients, with this positive effect appearing linked to the regulation of key growth-related genes (GHR, IGF, LPL, and FAS) and improvements in intestinal structure and function. Importantly, dietary supplementation with *M. oleifera* shown to effectively mitigate the negative effects of starvation stress, allowing fish to maintain enhanced growth performance even after periods without food, highlighting the potential of this plant to improve resilience in aquaculture systems **(**Elabd et al. 2019).

### Physiological impacts

#### Digestive enzyme activity

The inclusion of *M. oleifera* in fish diets shown to influence digestive enzyme activity, potentially enhancing nutrient utilization. Polyphenols, including phenolic acids and flavonoids found in Moringa, have demonstrated strong growth-promoting effects, by modulating digestive processes **(**Mahmoud et al. 2022). While specific studies on African catfish are limited research on other fish species suggests that *M. oleifera* supplementation can increase the activity of digestive enzymes such as amylase, lipase, and protease, leading to improved nutrient digestion and absorption. Puycha et al. (2017) demonstrated that the inclusion of *M. oleifera* leaves at 100 g could be utilized in the feed without negative effect on the growth performance, digestibility, and serum biochemistry.

#### Metabolic effects

*M. oleifera* demonstrates significant metabolic benefits in aquaculture species through its bioactive compounds, including flavonoids, phenolic acids, and alkaloids, which collectively improve various physiological processes in fish **(**Mali et al. 2022; Mushtaq et al. 2021). Studies have revealed that *M. oleifera* supplementation positively affects liver function, with Elabd et al. (2019) reporting significant reductions in alanine aminotransferase (ALT) and aspartate aminotransferase (AST) activities in Nile tilapia, attributing these hepatoprotective effects to the presence of quercetin, which has been extensively studied for its beneficial impact on liver enzymes. Also, benefits kidney functions of fish from *M. oleifera* supplementation, are evidenced by Emam et al. (2024), who observed that moderate doses (200 mg/kg) of *M. oleifera* aqueous extract (MOAE) significantly reduced blood urea nitrogen (BUN) and creatinine (Cr) levels in Nile tilapia, though interestingly, higher doses (400 mg/kg) produced opposite effects, highlighting the importance of appropriate dosing in fish feeds.

Regarding stress management, Moringa’s rich antioxidant profile, which includes total phenols (785.5 mg/100 g gallic acid equivalent), total flavonoids (257 mg/100 g quercetin equivalent), and total antioxidant capacity (1701.8 mg/100 g ascorbic acid equivalent), contributes to its stress-reducing properties in fish (Al-Taweel and Al-Anbari 2019). Shourbela et al. (2020) demonstrated that dietary MOAE supplementation significantly lowered cortisol levels in fish during pre-hypoxic conditions and helped reduce hypoxia-induced stress after exposure, while Khalil and Korni (2017**)** observed significant reductions in plasma cortisol levels in *C. carpio* fed *M. oleifera* compared to control groups. The anti-inflammatory and antioxidant properties of *M. oleifera*, attributed to compounds like niazimicin, niaziminin, and β-sitosterol **(**Islam et al. 2021), further enhancement of metabolic benefits by protecting fish against oxidative damage and supporting overall physiological homeostasis. These metabolic effects collectively establish *M. oleifera* as a valuable functional feed additive with considerable potential to support physiological processes and help fish cope with environmental stressors, contributing to improved aquaculture production and sustainability.

### Immunomodulatory properties

*M. oleifera* has demonstrated significant immunomodulatory effects in various fish species, particularly in African catfish (*C. gariepinus*), attributed to its exceptionally rich phytochemical profile. This profile contains high concentrations of bioactive compounds including polyphenols (total phenolics: 785.5 mg/100 g gallic acid equivalent), flavonoids (257 mg/100 g quercetin equivalent), alkaloids (niazimicin, niaziminin, β-sitosterol), and various other immunostimulatory phytochemicals that collectively enhance immune function **(**Mali et al. 2022). These compounds interact synergistically with the distinctive immune system of African catfish, which possesses specialized adaptations for survival in hypoxic environments, making this species particularly responsive to plant-based immunostimulant **(**Enzeline et al. 2022). The immunomodulatory capacity of *M. oleifera* in African catfish can be examined through four critical aspects: enhancement of innate immunity, modulation of adaptive immune responses, molecular signalling mechanisms, and comparative efficacy across fish species (Gaber et al. 2025).

#### Molecular analysis and sampling procedures

Comprehensive molecular analysis of immunomodulatory effects requires standardized sampling and analytical protocols **(**Gad et al. 2024; Kamble et al. 2024). Fish are fasted 24 h before sampling (AVMA 2020), followed by anaesthesia using MS-222 (Tricaine methanesulfonate, Sigma-Aldrich Cat. No. A5040) at 100 mg/L concentration (AVMA 2020). Blood collection utilizes caudal vein puncture with 1 ml heparinized syringes (BD Plastipak™) containing lithium heparin (Sigma-Aldrich, Cat. No. H0878) at 50 IU/ml blood **(**El-Kassas et al. 2020).

Target tissues for molecular analysis include liver, spleen, kidney, gills and skin (100–200 mg each), with RNA preservation using RNAlater solution (Thermo Fisher, Cat. No. AM7020) at −80 °C storage **(**Rohani et al. 2022; Sharker et al. 2024). Quality control measures include RNA integrity assessment using Agilent 2100 Bioanalyzer (Agilent Technologies) with RIN (RNA Integrity Number) ≥ 7.0 acceptable for qPCR analysis **(**Gaber et al. 2025).

Gene expression analysis utilizes Applied Biosystems StepOnePlus™ Real-Time PCR System with TaqMan probes for cytokine analysis (Thermo Fisher Custom Assays) and β-actin, GAPDH reference genes for normalization (Livak and Schmittgen 2001). Data analysis employs relative quantification using 2^(−ΔΔCt)^ method for accurate comparison between treatment groups **(**Rao et al. 2013).

#### Enhancement of innate immune defences

African catfish exhibit a robust innate immune system characterized by high baseline levels of non-specific defence mechanisms. The innate immune system provides the first line of defence against infections, encompassing physical barriers, cellular processes, and humoral components **(**Sahoo et al. 2021). In fish, the innate immune response is crucial initially because of the slow proliferation of lymphocytes and a limited antibody repertoire, which delays the adaptive immune response (Firdaus-Nawi and Zamri-Saad 2016**)**. Lysozyme activity, a crucial antimicrobial enzyme, shows significant enhancement in African catfish fed with *M. oleifera* supplements. This effect results from the high sensitivity of catfish mucosal lysozyme to plant-derived phenolic compounds such as quercetin and kaempferol found abundantly in *M. oleifera* **(**Mali et al. 2022). The phagocytic respiratory burst activity, another critical innate immune function, increases in Moringa-supplemented African catfish, indicating enhanced capacity of neutrophils and macrophages to generate reactive oxygen species for pathogen elimination.

Complement activation, a vital component of innate immunity, also shows remarkable enhancement in Moringa-supplemented African catfish. The mechanisms underlying these enhancements involve Moringa’s flavonoids (particularly quercetin and kaempferol) that upregulate the expression of pattern recognition receptors in African catfish, including Toll-like receptors (TLRs). This is particularly important as fish possess most of the primary and secondary lymphoid organs found in mammals, though with reduced complexity, potentially limiting the development of fully functional adaptive immune responses **(**Firdaus-Nawi and Zamri-Saad 2016**).**

#### Modulation of adaptive immune responses

The adaptive immune system of African catfish exhibits a unique response to Moringa supplementation, with studies showing a marked increase in total serum IgM levels among fish fed with *M. oleifera*_ supplements. This enhancement is credited to Moringa’s ability to promote B-lymphocyte proliferation and differentiation, particularly in African catfish, which contain a higher concentration of IgM-secreting cells in their head kidney compared to other farmed fish species **(**Sheng et al. 2024). Additionally, *M. oleifera* supplementation has a significant impact on the cytokine profiles of fish. Cytokines, which are secreted by both innate and adaptive immune cells, play a crucial role in cell signaling **(**Zou and Secombes 2016**).** g. Research has revealed that *M. oleifera* leads to considerable upregulation of pro-inflammatory cytokine genes such as IL-1β, TNF-α, IL-6, and IL-8 **(**Kalb et al. 2021), while moderately increasing the expression of anti-inflammatory cytokines like IL-10 and TGF-β **(**Gad et al. 2024). This balanced cytokine expression fosters an optimal immunostimulatory state that enhances pathogen clearance while minimizing tissue damage.

Further studies indicate that fish supplemented with *M. oleifera*_ aqueous extract exhibits significantly elevated levels of TNF-α and IL-1β compared to control groups. This aligns with findings that *M. oleifera* enhances the expression of pro-inflammatory cytokines, an effect likely tied to increased lymphocyte numbers and the high concentration of isothiocyanates in *M. oleifera* leaves. TNF-α plays a vital role in regulating inflammation and stimulating immune responses by enhancing the phagocytic activity of fish leukocytes El-Kassas et al. (2020). Furthermore, TNF-α is a pivotal cytokine within the network of pro- and anti-inflammatory cytokines and is well-documented to stimulate its production as well as that of other cytokines like IL-1, IL-6, and IL-8 **(**Rahman and McFadden 2006**).** TNF-α is also one of the first immune-related genes and is crucial in modulating inflammation while stimulating immune function by boosting the phagocytic activity of fish leukocytes **(**Rohani et al. 2022).

The expression of major histocompatibility complex (MHC) class II molecules, crucial for antigen presentation to T lymphocytes, also increases significantly in Moringa-supplemented African catfish, enhancing T-cell activation and subsequent cell-mediated immunity against intracellular pathogens. Together, these adaptive immune enhancements significantly improve disease resistance in African catfish, with challenge studies demonstrating higher survival rates against bacterial infections in Moringa-supplemented fish compared to controls.

#### Molecular signalling mechanisms and cellular pathways

The immunomodulatory effects of *M. oleifera* in fish operate through specific molecular mechanisms that have elucidated through advanced immunological techniques. Multiple signalling pathways significantly activated by Moringa’s bioactive compounds, particularly isothiocyanates present in *M. oleifera* leaf extract (MOLE). These compounds include (α-L-rhamnopyranosyloxy) benzyl glucosinolate (glucomoringin) and various derivatives including [(2′-O-acetyl-α-L-rhamnosyloxy) benzyl] glucosinolate, [(3′-O-acetyl-α-L-rhamnosyloxy) benzyl] glucosinolate, and [(4′-O-Acetyl-α-L-rhamnosyloxy) benzyl] glucosinolate **(**Mali et al. 2022).

Various researchers have highlighted the hypoglycaemic, anti-inflammatory, anti-cancer, and antioxidant effects of *M. oleifera* glucosinolates and isothiocyanates, observed through various in vitro and vivo studies **(**Lopez-Rodriguez et al. 2020). In fish, these compounds appear to modulate key transcription factors regulating immune responses, including nuclear factor-κB (NF-κB), an expert transcription factor regulating genes involved in inflammation and immunity. The antioxidant defence system is simultaneously enhanced with *M. oleifera* supplementation, upregulating the expression of antioxidant enzymes in African catfish immune cells. Research found that superoxide dismutase (SOD) activity decreased in the liver, gills, and muscles of *Cirrhinus mrigala* fish exposed to arsenic only. However, in fish-fed diets supplemented with 2% and 4% *M. oleifera* extract along with arsenic, enzyme activity improved, and oxidative stress reduced in these tissues **(**Azhar et al. 2023). This antioxidant enhancement protects immune cells from oxidative damage during respiratory burst activity, maintaining optimal immune function during heightened antibacterial responses and creating a comprehensive molecular foundation for enhanced immunocompetence.

#### Comparative efficacy and practical applications

The immunostimulatory effects of *M. oleifera* in African catfish exhibit a distinctive dose-response relationship that differentiates them from other fish species and offers practical guidance for aquaculture applications. Studies indicate an optimal immunostimulant achieved at moderate supplementation levels, with diminishing returns observed at higher concentrations and potential immunosuppression at extremely high doses due to anti-inflammatory compounds like quercetin becoming inhibitory. Comparative analysis reveals that African catfish exhibit a stronger immunostimulatory response to *M. oleifera* supplementation than other fish species. This may be due to their higher density of pattern recognition receptors in mucosal surfaces that interact with *M. oleifera* phytochemicals, greater sensitivity of signalling pathways to plant-derived compounds, and unique adaptations in their antioxidant systems that synergize with Moringa’s radical-scavenging components. These species-specific responses translate to practical benefits in aquaculture settings, with Moringa-supplemented African catfish showing significant improvements in production parameters including higher specific growth rate, better feed conversion ratio, and Lower cumulative mortality compared to standard feeds. Incorporating up to 30% *M. oleifera* leaf meal in the diet is both feasible and beneficial for maximizing catfish growth, suggesting this level is most effective for optimal performance **(**Adekilekun et al. 2022).

Additionally, the environmental sustainability of *M. oleifera* as an immunostimulant is noteworthy, as it reduces reliance on synthetic antibiotics and chemotherapeutants, potentially mitigating antimicrobial resistance development and environmental contamination in aquaculture settings. Antibiotics are crucial in preventing infections, but their overuse can lead to antibiotic resistance in bacteria, making them less effective against future infections **(**Okeke et al. 2022). To reduce antibiotic dependence in aquaculture, natural alternatives such as *M. oleifera* present promising solutions. Studies have demonstrated the antibacterial properties of *M. oleifera* in fish aquaculture. Parveen et al. (2021) found the highest levels of disease-causing bacteria in the intestines of control fish compared to those fed diets supplemented with varying concentrations of *M. oleifera* leaves. The control fish exhibited significant presence of *Escherichia coli*, *P. aeruginosa*, *Shigella*, and *Salmonella*, while Moringa-supplemented fish demonstrated substantial reduction in the growth of these bacteria. These findings collectively establish *M. oleifera* as a particularly effective immunostimulant for African catfish aquaculture, with species-specific efficacy that offers considerable benefits for sustainable fish farming practices.

### Blood parameters

#### Haematological indices

*M. oleifera* supplementation exerts diverse effects on haematological parameters in various fish species, with Sharker et al. (2024) finding that fish fed with a 10% *M. oleifera* leaf meal (MOLM) diet showed significantly higher erythrocyte count compared to the control fish in *Heteropneustes fossilis*, while Emam et al. (2024) reported that Nile tilapia fed a diet supplemented with 200 mg/kg MOAE demonstrated a significant improvements in RBC count, though higher doses (400 mg/kg) led to significant decreases **(**Bbole et al. 2016). The bioactive compounds present in Moringa, including phenolics (785.5 mg/100 g gallic acid equivalent) and flavonoids (257 mg/100 g quercetin equivalent), likely contribute to these haematological effects through their antioxidant properties (Al-Taweel and Al-Anbari 2019), protecting red blood cells from oxidative damage and potentially stimulating erythropoiesis **(**Oghenochuko and Mshelbwala 2021**).**

Haemoglobin levels have also shown positive responses to appropriate *M. oleifera* supplementation, with Faisal et al. (2024) reporting that fish fed a 10% MOLM diet exhibited the highest haemoglobin level (8.52 g/100 mL) compared to other inclusion levels, while Arsalan et al. (2016) similarly found that *Labeo rohita* fingerlings on a 10% MOLM diet had the highest haemoglobin levels (8.91 g/100 mL), though contrasting results were observed by Moustafa and Assem (2024**)**, who noted no significant changes in haemoglobin content of *O. niloticus* fed on a *M. oleifera* seed diet at any concentration compared to the control. Haematocrit values also respond to *M. oleifera* supplementation, with Arsalan et al. (2016) finding the highest PCV value (28.01%) in *L. rohita* fingerlings fed a 20% MOLM-based diet, though a decreasing trend was observed in fish fed 30–40% MOLM diets, suggesting that excessive inclusion levels may have diminishing returns or potentially adverse effects on blood parameters. The rich mineral profile of *M. oleifera* leaves, which includes iron (28.2 mg/100 g), calcium (1.322–2.645 g/100 g), and magnesium (368 mg/100g) **(**Sultana 2020**)**, likely plays a significant role in supporting erythropoiesis and haemoglobin synthesis in fish, particularly through the high iron content, which is essential for hemoglobin formation and oxygen transport.

#### Biochemical parameters

*M. oleifera* supplementation influences various biochemical parameters in fish, with particularly notable effects on glucose metabolism, as Moustafa and Assem (2024**)** reported significantly lower serum glucose levels in *O. niloticus* fed *M. oleifera* seed diet at certain concentrations compared to the control, while glucose levels decreased significantly in fish-fed *M. oleifera* leaf diet at all concentrations. These hypoglycaemic effects align with Elabd et al. (2019), findings who observed that in Moringa-supplemented groups of Nile tilapia, glucose levels significantly decreased throughout the experiment, which was attributed to the antioxidant properties of *M. oleifera* potentially enhancing insulin activity, as well as the presence of glucosinolates and their hydrolysis products in *M. oleifera* leaves that have demonstrated hypoglycaemic effects through in vitro and in vivo studies **(**Lopez-Rodriguez et al. 2020).

*M. oleifera* supplementation can influence total protein levels, as demonstrated by Abbas and El-Badawi (2014**)**, who observed a decline in total protein levels in *O. niloticus* exposed to *M. oleifera* seed extract under both acute and chronic conditions. However, this impact may vary depending on the bioactive compounds present in different *M. oleifera* formulations and their concentrations. Additionally, the lipid profile of fish appears responsive to *M. oleifera* supplementation, with El-Kassas et al. (2020) reporting enhanced blood lipid levels in Nile tilapia fingerlings fed diets containing *M. oleifera* leaf powder. This suggests a positive influence on lipid metabolism, potentially driven by pharmacologically active alkaloids such as niazimicin, niaziminin, and β-sitosterol, which can modulate metabolic pathways related to lipid regulation **(**Mali et al. 2022). The diverse effects of *M. oleifera* on fish blood biochemical parameters highlight the intricate interaction between its array of bioactive compounds and fish physiology. This underscores the necessity of identifying optimal inclusion levels for various fish species to maximize benefits while mitigating potential risks. Specifically, compounds like benzyl isothiocyanate found in *M. oleifera* have been shown to induce intracellular reactive oxygen species, which may exhibit dose-dependent effects on cellular metabolism and physiological functions **(**Islam et al. 2021).

### Disease resistance

*M. oleifera* has demonstrated significant potential in enhancing disease resistance in various fish species, including African catfish. This enhanced resistance attributed to the plant’s immunostimulatory, antimicrobial, and antioxidant properties.

#### Antimicrobial mechanisms

*M. oleifera* possesses remarkable antimicrobial properties against major fish pathogens through its diverse phytochemical profile, with extracts from the leaf, flower, root bark, and stem bark demonstrating effective antimicrobial and anthelmintic activities against a wide range of fish pathogens **(**Islam et al. 2021). The antimicrobial compound pterygospermin, identified in *M. oleifera* leaves and flowers by Das et al. (1957) and Rao et al. (2001), exhibits powerful antimicrobial activity against *A. hydrophila*, one of the most economically devastating bacterial pathogens in freshwater aquaculture, directly inhibiting its proliferation through disruption of bacterial cell walls **(**Momin and Memiş 2023**).** Against *P. aeruginosa*, a significant opportunistic pathogen in both freshwater and marine aquaculture, ethanolic extracts of *M. oleifera* seeds, leaves, and flowers demonstrate particularly strong antimicrobial activity, attributed to their high content of phenolic compounds that disrupt bacterial membrane integrity, with in vitro studies indicating that Moringa-derived compounds directly inhibit the production of pyocyanin and other virulence factors essential for Pseudomonas pathogenicity **(**Jayawardana et al. 2015).

The plant shows remarkable effectiveness against various *Vibrio* spp, primary pathogens in mariculture, with research consistently demonstrating that *V. cholerae* is highly susceptible to crude leaf powder and extracts of *M. oleifera*, as the antimicrobial compounds including alkaloids, flavonoids, phenolics, and tannins disrupt essential *Vibrio* metabolic pathways and cell membrane integrity **(**Jayawardana et al. 2015). While specific studies on Moringa’s efficacy against *Edwardsiella* are limited in the literature, the plant’s broad-spectrum antibacterial properties suggest significant potential against this gram-negative pathogen that causes severe economic losses in catfish, eel, and tilapia farming, likely functioning through similar mechanisms as observed with other gram-negative bacteria, disrupting cell membranes and inhibiting essential metabolic pathways **(**Islam et al. 2021). Moringa’s ability to modulate gut microbiota has been demonstrated by Parveen et al. (2021), who observed higher levels of disease-causing bacteria including *E. coli*, *P. aeruginosa*, *Shigella*, and *Salmonella* in the intestines of fish in control groups compared to those fed diets supplemented with *M. oleifera* leaves, with significant reductions in pathogen populations in the Moringa-supplemented groups, potentially due to bioactive compounds isolated from ethanol extracts that possess diverse pharmacological properties **(**Momin and Memiş 2023**).**

#### Pathogen challenge studies

*M. oleifera* supplementation has consistently demonstrated enhanced protection against various fish pathogens in challenge studies, with Mbokane and Moyo (2018**)** conducting a comprehensive examination of Moringa-based diets in *Oreochromis mossambicus* pre- and post-challenge with *A. hydrophila*, finding that supplementation at 9–12% inclusion levels significantly enhanced survival rates while maintaining immune function during pathogen exposure **(**Momin and Memiş 2023**).** Kamble et al. (2024) reported that diets supplemented with 0.5% *M. oleifera* leaf extract (MLE) significantly improved survival rates of Nile tilapia infected with *Streptococcus agalactiae* Biotype 2 compared to control groups, with the enhanced resistance attributed to the quercetin-rich composition of *M. oleifera* leaves, as identified by Bennett et al. who reported chlorogenic acid, gallic acid, kaempferol, and glycoside presence in *M. oleifera* leaves, compounds known for their antimicrobial properties **(**Islam et al. 2021).

Studies with seabream have shown that 5–10% *M. oleifera* leaf inclusion in diets significantly enhances mucosal immunity, providing a crucial first line of defence against *Vibrio* infections, which typically enter through mucosal surfaces **(**Mansour et al. 2020). In African catfish studies, *M. oleifera* supplementation has demonstrated not only improved growth parameters but simultaneously enhanced resistance to Aeromonas infections, making it particularly valuable in semi-intensive aquaculture systems where Aeromonas outbreaks are common **(**Ayoola et al. 2013). For marine species particularly susceptible to vibriosis, *M. oleifera* supplementation has emerged as a promising natural alternative to antibiotics, helping to address growing concerns about antimicrobial resistance in aquaculture **(**Momin and Memiş 2023**).** The water-soluble, cationic proteins in *M. oleifera* seeds that function as effective natural coagulants for water treatment may also contribute to disease resistance by improving water quality parameters in aquaculture systems, thereby reducing pathogen loads and environmental stress on fish **(**Islam et al. 2021).

#### Survival rates

*M. oleifera* supplementation significantly enhances survival rates in aquaculture species challenged with various bacterial pathogens, with multiple studies demonstrating its functional properties including water absorption capacity (158.00-415.50%), foaming capacity (28.30–117.65 mL/L), and foaming stability (333.33–1000 mL/L) potentially contributing to improved water quality parameters that support fish health and survival during pathogen exposure **(**Sultana 2020**).** Optimal inclusion levels for maximum protection against bacterial pathogens have established through extensive research, with 10–15% *M. oleifera* inclusion recommended for carnivorous fish and 15–20% for omnivorous species providing the best prophylactic effects against Pseudomonas infections while maintaining growth performance **(**Momin and Memiş 2023**).** For *A. hydrophila* resistance, studies utilizing various forms of *M. oleifera* supplementation at appropriate levels consistently show improved immunity and enhanced survival rates in Nile tilapia and other commercially important species **(**Abidin et al. 2021).

During stress conditions, which often predispose fish to bacterial infections, Moringa’s anti-stress properties, as evidenced by reduced cortisol levels in supplemented fish **(**Shourbela et al. 2020; Khalil and Korni 2017**)**, can be attributed to its diverse bioactive compounds including alkaloids, which possess notable pharmacological properties such as anti-inflammatory activities that help fish better cope with pathogen challenges that typically exploit immunocompromised states **(**Mali et al. 2022). The enhanced survival rates following exposure to pathogens like *Vibrio* and *Aeromonas* can be linked to Moringa’s traditional medicinal applications, as it has been used to treat many ailments such as infection, inflammation, and to promote wound healing, properties that collectively support fish health during bacterial disease challenges **(**Sultana 2020**).** For African catfish, where *Edwardsiella* infections are particularly problematic, the recommended *M. oleifera* inclusion levels of 15–20% **(**Momin and Memiş 2023; Idowu et al. 2017) provide optimal protection while supporting growth performance, highlighting the plant’s versatility in managing multiple pathogen types, as noted the increasing interest in utilizing natural alternatives to reduce antibiotic dependence in aquaculture positions *M. oleifera* as an exceptional candidate for sustainable disease management in fish farming, supported by extensive traditional knowledge of its medicinal properties spanning the Ayurvedic and Unani systems, which have long recognized its diverse therapeutic applications for treating various bacterial diseases **(**Islam et al. 2021).

## *M. oleifera* seeds: applications and toxicity concerns

### Water clarification properties

*M. oleifera* seeds have garnered significant attention for their remarkable water clarification properties, offering valuable applications in aquaculture systems where water quality management remains a critical concern **(**Abbas and El-Badawi 2014**).** The water purification ability of *M. oleifera* seeds is primarily attributed to the presence of cationic proteins that function as natural coagulants through several mechanisms, including charge neutralization, where positively charged proteins neutralize negative charges on suspended particles in water, reducing electrostatic repulsion and facilitating aggregation **(**Desta and Bote 2021**).** These protein molecules further enhance flocculation through a bridging mechanism by adsorbing onto multiple particle surfaces simultaneously, while at higher concentrations, they can form mesh-like structures that sweep through water, capturing and removing suspended particles through a process known as sweep coagulation **(**Gopalakrishnan et al. 2016).

The efficacy of *M. oleifera* seed extract in reducing water turbidity has been well-documented, with studies demonstrating turbidity reduction of 95–99% in highly turbid water, performance that is comparable to or exceeds conventional chemical coagulants such as aluminium sulphate **(**Abd El-Hack et al. 2022; Muhd et al. 2024). Optimal dosage for turbidity reduction typically ranges from 10 to 200 mg/L, depending on initial turbidity levels and water characteristics, making it adaptable to various aquaculture systems where water clarity is essential for photosynthesis by beneficial algae, efficient feeding, and overall system health **(**Oyeyinka and Oyeyinka 2018**).** Beyond turbidity reduction, *M. oleifera* seed extracts have demonstrated considerable potential for removing heavy metals from water through their metal binding capacity, as the proteins and other compounds contain functional groups such as carboxyl, hydroxyl, and amino groups that effectively bind to heavy metal ions **(**Hsu et al. 2024).

Studies have confirmed the ability of *M. oleifera* seed extracts to remove various heavy metals including lead, cadmium, copper, zinc, and chromium—contaminants particularly harmful to fish in aquaculture systems—primarily through adsorption, ion exchange, and complexation mechanisms (Nwagbara et al. 2022).

### Antinutritional factors

Despite their beneficial water clarification properties, *M. oleifera* seeds contain several antinutritional factors such as phytates, saponins, tannins, oxalates, and alkaloids that can interfere with nutrient utilization when consumed by fish or when seed extracts added to water at high concentrations **(**León-López et al. 2020). Tannins present in *M. oleifera* seeds can bind to proteins and enzymes, reducing their digestibility and activity while potentially irritating the digestive tract and interfering with mineral absorption in fish **(**Muhd et al. 2024). Phytic acid represents another significant antinutritional factor in *M. oleifera* seeds that can bind to essential minerals including calcium, zinc, iron, and magnesium, reducing their bioavailability and potentially leading to mineral deficiencies in cultured fish **(**Abd El-Hack et al. 2022).

Saponins found in *M. oleifera* seeds can adversely affect membrane permeability and potentially cause haemolysis of red blood cells in fish while imparting a bitter taste that may reduce feed palatability and consequently impact feed intake and growth performance **(**Oyeyinka and Oyeyinka 2018**).** Glucosinolates, while possessing beneficial properties at certain concentrations, can become goitrogenic at elevated levels, potentially affecting thyroid function and related metabolic processes in fish (Khalil and Korni 2017). Additionally, oxalates present in *M. oleifera* seeds can form insoluble complexes with calcium, potentially leading to calcium deficiency, particularly in juvenile fish where rapid skeletal development requires adequate calcium availability **(**Grosshagauer et al. 2021).

The concentration and activity of these antinutritional factors can vary significantly based on factors including seed maturity, processing methods, extraction techniques, and environmental growth conditions of the parent plant **(**Gopalakrishnan et al. 2016). Various processing methods such as heat treatment, fermentation, and soaking have been investigated for their effectiveness in reducing antinutritional factors in *M. oleifera* seeds, with thermal treatments like boiling demonstrating promise in inactivating certain compounds while preserving beneficial properties, though complete elimination of all antinutritional factors remains challenging **(**Muhd et al. 2024).

### Toxicity studies in fish

Research has documented that exposure to high concentrations of *M. oleifera* seed extracts can induce acute toxic effects in fish, particularly affecting haematological parameters **(**Abbas and El-Badawi 2014**).** Both acute and chronic exposure to *M. oleifera* seed extract in *O. niloticus* has been reported to cause decreased red blood cell count, haemoglobin, haematocrit, and mean corpuscular haemoglobin concentration, while simultaneously increasing white blood cell count, and mean corpuscular volume, indicating potential anaemia and immune system activation **(**Muhd et al. 2024).

Behavioural observations in toxicity studies have documented abnormal swimming patterns, reduced activity levels, and increased opercular movement in fish exposed to high concentrations of *M. oleifera* seed extracts, suggesting potential neurotoxic effects that warrant further investigation regarding underlying mechanisms **(**Kavitha et al. 2012). Chronic exposure studies show that some processing methods can reduce the adverse effects of *M. oleifera* seed meal. However, certain forms still induce histopathological changes in fish tissues. Muhd et al. (2024) reported that fish fed diets containing ethanol-extracted, raw, autoclaved, or toasted *M. oleifera* seed kernel meal exhibited various histological alterations in the liver and kidney, including severe inflammation and necrosis. Interestingly, the boiled *M. oleifera* seed kernel meal did not cause any significant negative histological effects.

Limited research suggests potential reproductive impacts of long-term *M. oleifera* seed exposure, while growth inhibition at high inclusion levels has been reported across multiple fish species, likely attributable to antinutritional factors and other bioactive compounds (de Santana et al. 2017). Establishing safe inclusion levels for *M. oleifera* seeds in fish diets requires consideration of species-specific sensitivities, fish developmental stage, processing methods applied to the seeds, and overall dietary composition, with further research needed to fully elucidate dose-dependent relationships between *M. oleifera* seed components and fish physiological responses **(**Abd El-Hack et al. 2022).

## Conclusion

*M. oleifera* holds significant promise as a versatile and sustainable resource for African catfish aquaculture. This review highlights its potential to enhance growth, improve physiological functions, boost immune responses, and strengthen disease resistance when included in catfish diets at optimal levels (5–30%). Additionally, its antimicrobial properties against major aquaculture pathogens, such as *A. hydrophila*, *P. aeruginosa*, and various *Vibrio* spp, offer a natural alternative to antibiotics, addressing concerns about antimicrobial resistance. Beyond nutritional benefits, *M. oleifera* seeds contribute to water clarification through their cationic proteins, which act as natural coagulants, though careful attention is needed to mitigate toxicity risks from antinutritional factors at high concentrations. Economically, incorporating *M. oleifera* products into catfish feed has proven highly cost-effective, with studies showing feed cost reductions of up to 67.6% when processed *M. oleifera* seed meal replaces conventional fishmeal. Coupled with its widespread availability in tropical and subtropical regions, this economic advantage positions *M. oleifera* as an excellent candidate for advancing sustainable aquaculture, particularly in resource-limited areas.

Future research should focus on standardizing processing methods to minimize antinutritional factors, establishing species-specific optimal inclusion rates for various life stages, conducting long-term studies on reproductive effects, and exploring potential synergistic effects when *M. oleifera* combined with other natural feed additives. The development of commercial Moringa-based feed formulations specifically optimized for farmed fish represents a significant opportunity for the aquaculture industry. As aquaculture continues to expand globally, the integration of *M. oleifera* as both a feed additive and water treatment agent offers a promising pathway toward more sustainable, economically viable, and environmentally responsible fish production systems that can contribute significantly to global food security.

## Data Availability

No datasets were generated or analysed during the current study.
